# Volume transmission of beta-endorphin via the cerebrospinal fluid; a review

**DOI:** 10.1186/2045-8118-9-16

**Published:** 2012-08-10

**Authors:** Jan G Veening, Peter O Gerrits, Henk P Barendregt

**Affiliations:** 1 Department of Anatomy (109), University Medical Center St. Radboud, PO Box 9101, 6500, HB, Nijmegen, the Netherlands; 2 Department of Psychopharmacology, UIPS, University of Utrecht, Utrecht, the Netherlands; 3 Department of Neuroscience, section Anatomy, University Medical Center Groningen, University of Groningen, Groningen, the Netherlands; 4 Faculty of Science, Radboud University, Nijmegen, the Netherlands

**Keywords:** β-endorphin, Pro-opio-melanocortin, Cerebrospinal fluid, Volume transmission, Arcuate nucleus of the hypothalamus, Behavior

## Abstract

There is increasing evidence that non-synaptic communication by volume transmission in the flowing CSF plays an important role in neural mechanisms, especially for extending the duration of behavioral effects. In the present review, we explore the mechanisms involved in the behavioral and physiological effects of β-endorphin (β-END), especially those involving the cerebrospinal fluid (CSF), as a message transport system to reach distant brain areas. The major source of β-END are the pro-opio-melano-cortin (POMC) neurons, located in the arcuate hypothalamic nucleus (ARH), bordering the 3^rd^ ventricle. In addition, numerous varicose β-END-immunoreactive fibers are situated close to the ventricular surfaces. In the present paper we surveyed the evidence that volume transmission via the CSF can be considered as an option for messages to reach remote brain areas. Some of the points discussed in the present review are: release mechanisms of β-END, independence of peripheral *versus* central levels, central β-END migration over considerable distances, behavioral effects of β-END depend on location of ventricular administration, and abundance of mu and delta opioid receptors in the periventricular regions of the brain.

## Introduction

There is increasing evidence that non-synaptic communication by volume transmission in the flowing CSF plays an important role in neural mechanisms, especially for extending the duration of behavioral effects [1-4]. Beta-Endorphin (β-END) is a neuropeptide, produced by pro-opio-melanocortin (POMC) neurons as well as by pituitary cells mainly located in the intermediate lobe [5,6], by cleavage from a larger precursor molecule, beta-lipotropin. β-END is its C-fragment (containing the amino acids 61–91) and was characterized by Guillemin *et al*, in 1977 [7,8], in combination with its sister peptides, α-melanocyte-stimulating hormone (α-MSH), adrenocorticotropic hormone, (ACTH) and other substances [9-13]. The molecular weight of β-END is 3465 g/mol.

The behavioral effects of β-END were soon recognized and vary from prolonged muscular rigidity [14] to general arousal [15]. More specifically, β-END was shown to play a role in several kinds of behavior, like feeding [16-18], sexual behavior [19,20], learning processes [21,22], reward [23,24], pain-regulating mechanisms [25-31], as well as in a variety of physiological functions such as cardiovascular regulation [32,33] and stress responses [12,34-37].

Interestingly, β-END is produced in the pituitary for release into the peripheral systemic circulation, and by hypothalamic POMC neurons for release inside the central nervous system (CNS). Since it has been observed that peripheral administration of β-END does not necessarily induce the same effects as intracerebroventricular (icv) administration, this suggests the existence of two functionally different β-END systems, one for the central effects and one for the peripheral effects. The present review explores the existence of a special central and brain-directed β-END system and the possibility that the cerebrospinal fluid (CSF) plays a special role in the propagation of these brain-directed β-END messages.

The evidence discussed in the present review has led to the conclusion that CSF-levels of β-END are not a reflection of the peripheral levels, but are controlled and regulated by separate inputs and by specific mechanisms that are functionally separate from the pituitary release mechanisms involved in the plasma levels. This conclusion does not necessarily indicate that central CSF and peripheral plasma levels of β-END are totally unrelated. Peripheral β-END may be able to access the CNS from the periphery via the circumventricular organs (CVO’s) lacking in a blood–brain barrier (BBB), and the choroid plexus [38,39]. In addition, the spinal cord seems to be accessible for entrance of blood born proteins [40] and for reentrance of proteins circulating in the surrounding arachnoid space [41-43]. In the other direction, a saturable transporter mechanism, P-glycoprotein**,** from brain to blood makes it possible that CSF peptides like β-END can gain access to peripheral systems at the brain capillaries [44-46]. These and other efflux mechanisms most probably serve a modulatory purpose to integrate central/behavioral and peripheral responses.

The flowing CSF serves as a medium to transport neuropeptides or other substances to distant receptive brain areas. This type of transport has been described as long-distance volume transmission (VT) [1-3,39,47-56]. For a few neuropeptides, the evidence favoring such a message function for the CSF has been reviewed in more detail: for vasopressin, corticotropin releasing hormone (CRH) [48,49], and for oxytocin (OT) [4]. For other substances, like melatonin, gonadotropin-releasing hormone as well as for factors influencing food intake, the evidence is convincing that substances, released into the ventricular system at a specific site, exert their effects at a different brain location, arriving there by moving with the flow of the CSF [57-66].

On the basis of the evidence presented here, we propose the following: There are two functionally different systems for the release of β-END, one for the peripheral effects via the systemic circulation and one directed to the central nervous system. The latter system uses synaptic communication and additional volume transport mechanisms provided by the flowing CSF. It is on the second part of this hypothesis that our present paper is focused.

## Sources of β-END

### POMC neurons in the hypothalamic arcuate nucleus (ARH)

**Figure 1:**
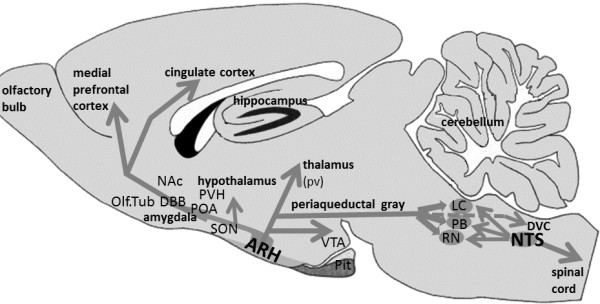
**Diagram showing the opiocortin projections from the arcuate nucleus of the hypothalamus.** The arcuate nucleus (ARH) contains most β-END cell bodies and is located in the mediobasal hypothalamus, at both sides of the 3^rd^ ventricle (not shown). Extensive opiocortin projections arise from the ARH and extend in rostral, cortical, hypothalamic and caudal brainstem directions. The most caudal projections extend into the dorsal vagal complex (DVC), comprising the area postrema, the dorsal vagal nucleus and the nucleus of the solitary tract (NTS). Limbic regions predominate as target areas for the ARH projections. In the caudal brainstem, in the NTS itself, another group of β-END cells has been detected. Their opiocortin fibers project mainly to brainstem areas as well as down the spinal cord. A number of brainstem areas, involved in numerous autonomic functions, receive a double innervation from both the ARH and the NTS: locus coeruleus (LC), the parabrachial region (PB) and several raphe nuclei (RN). Abbreviations: NAc: nucleus accumbens; Olf. Tub: olfactory tubercle; DBB: diagonal band nuclei; POA: preoptic area; PVH: paraventricular hypothalamic nucleus; SON: supraoptic hypothalamic nucleus; pv: periventricular thalamus; Pit: pituitary; VTA: ventral tegmental area.

Numerous immunocytochemical as well as *in situ* hybridization studies have confirmed the existence of a main population of β-END-immunoreactive (IR) neurons in the mediobasal hypothalamic region, most of them located in the arcuate hypothalamic nucleus (ARH) [67-73] (Figure 1). These neurons have been described as pro-opio-melanocortin (POMC) neurons because in these neurons a large precursor molecule (POMC) is cleaved into smaller peptides, like ACTH, α-MSH and β-END [11,71]. At the electron microscope level, β-END IR processes penetrate the ependymal layer of the basal hypothalamic ventricular wall as well as the pia mater overlying the ventral surface of the hypothalamus [73]. The axons traverse the sub-ependymal layers and show many varicosities, local swellings containing numerous vesicles but without synaptic specializations, suggesting local non-synaptic release mechanisms [11,68,73-76]. Also, in *Xenopus*, β-END neurons have been described as contacting the CSF directly [77,78]. These contacts make it possible for the hypothalamic β-END cells to release their contents into either the CSF of the 3^rd^ ventricle or of the subarachnoid space [79], bordering the ARH ventrally. Interestingly, many POMC neurons also participate in an intrinsic local network resulting in many POMC-POMC synapses inside the ARH [80]. Such contacts may synchronize the activities of the POMC neurons to integrate or coordinate as a functional unit, and this mechanism may regulate the amount of β-END released into the CSF.

The process of cleaving POMC molecules, followed by additional processing during axonal transport (see below), controlled release and extrinsically controlled levels of receptors (see below), provides the POMC system with a high degree of plasticity. We mention a few aspects: Firstly, while ACTH, β-END and α-MSH are fully co-localized in the ARH POMC neurons [69,70,72], their cellular concentrations are rather different. Secondly, ACTH and β-END are present in the same secretion granules [81] in about equimolar quantities, but the amount of α-MSH is at least 4 times higher in the cell bodies and up to 15 times higher in some terminal fields [70], suggesting that further processing of these neuropeptides occurs during axonal transport [70,82]. Therefore, it is improbable that the POMC derivatives will be secreted in a fixed balance over all trajectories or terminal fields [83]. In addition, Swanson *et al*[84-86] have shown that in paraventricular hypothalamic neurons, the balance between co-localized neuropeptides is under regulatory control, and can be disturbed by external factors like manipulation of the pituitary-adrenal axis or gonadectomy [84,86-88]. Such plastic fluctuations may lead to considerable variations in connectivity and to serious disturbances in the effects of activity in specific neuronal networks. Since similar changes seem to occur in the β-END projections to the supraoptic nucleus [89], these mechanisms appear to work in the POMC system also.

The extrinsic projections of the hypothalamic POMC neurons, also known as opiocortin projections, have been mapped extensively [68,76,90-96]. Their trajectories and destinations extend from rostral telencephalic regions, like the olfactory tubercle and diagonal band nuclei, to caudal brainstem areas like the ambiguous and lateral reticular nuclei [11,67,71].

There are a number of aspects of the POMC circuitry that deserve special attention***.*** There is a prevalence of POMC projections in brain areas such as the amygdala (central and medial nuclei), hypothalamus, periventricular thalamic nuclei and the periaqueductal gray (PAG). In the hypothalamus the densest innervations are provided to the parvocvellular, paraventricular, preoptic, periventricular and arcuate nuclei, which are all involved in anterior pituitary functions, via the median eminence [70]. The relationship between the paraventricular and supraoptic hypothalamic nuclei, including their magnocellular parts, has been studied in detail [89,94,97-101] and indicate possible modulatory effects of ACTH or β-END on the peripheral release of vasopressin or oxytocin (OT). A remarkable co-distribution has been recognized between opiocortin fibers and the corticotrophin-releasing factor (CRF)-immunoreactive fibers [67,102], suggesting a specific role of β-END on the effects of activation of the hypothalamus-pituitary-adrenal (HPA) axis, involving stress. In addition, the catecholaminergic cells, like noradrenergic neurons in the locus coeruleus as well as serotonergic neurons in the pontine raphe nuclei [67,93] receive a dense POMC innervation which suggests a regulatory involvement of β-END in a wide variety of brain functions. The more so as these brainstem regions receive additional opiocortin fibers from the neurons located in the lower brainstem (see below).

Studies combining retrograde tracers with POMC staining techniques have shown that subpopulations of the POMC neurons project to different destinations [103]. The β-END innervation of the ependymal and subependymal layers surrounding the ventricular system is extremely dense at some locations but varies considerably [67,68,70,73-76,93,95,104]. Despite the common origin of the POMC-derived neuropeptides, the relative densities of the ACTH, β-END and α-MSH fibers along the ventricular walls also varies considerably [70].

### POMC neurons in the caudal brainstem

In 1983 an additional group of opiocortin neurons was described in the caudal brainstem, within the commissural division of the nucleus tractus solitarius (NTS) [67,105]. These neurons project rostrally towards several pontine and medullary regions that also receive projections from the ARH. Apparently, autonomic brainstem regions like the parabrachial nucleus and locus coeruleus are provided with a double opiocortin innervation originating from both the arcuate nucleus and the caudal brainstem [106]. Other projections from this caudal group descend into the spinal cord, via the (dorso-)lateral funiculus to terminate around the central canal, and may be involved in the modulation of pain transmission [107].

### Pituitary: the source for peripheral release

In addition to the POMC neurons in the brain, the pituitary contains large numbers of POMC-producing cells. These cells are located in the intermediate as well as in the anterior lobe. Interestingly, however, the processing of the large POMC molecule seems to vary in different parts of the pituitary. In the anterior pituitary cells, ACTH is one of the main products of the POMC fragmentation, while in the intermediate lobe β-END and α-MSH predominate as the main fragments of POMC processing [11,13,36,108-110]. The paucity of vessels in the intermediate lobe [111,112] raises questions about the route used after cellular release, the more so as the human pituitary does not have a distinct pars intermedia, which is present in the whale, elephant and several other mammals [113]. Accordingly, only very low concentrations of α-MSH can be detected in the adult human pituitary gland [113,114]. These questions, related to the specific release mechanisms of the pituitary, are, however, beyond the scope of our present review.

In summary, there are three sources of POMC and its derivatives including β-END: the ARH, NTS and pituitary. The first two are directed towards the CNS including the spinal cord, the last one towards the systemic circulation and peripheral organs.

## Central and peripheral β-END are regulated differentially

### Peripheral changes in β-END only minimally affect CSF β-END

Early experiments showed that β-END in brain changed very little, or not at all, after hypophysectomy, while the peripheral concentration decreased dramatically and it was concluded that brain and peripheral β-END were regulated independently [8,110,115]. To study the differential regulatory mechanisms in more detail, the relative concentrations of β-END in CSF and plasma were determined. The CSF/plasma ratio ranged from 1.5 to 3 in rodents, [115-118] with an occasional exception [119], and up to 10 in humans [12,113,115-128], also with an occasional exception [129]. These variations appeared to be due to the diversity of methods and experimental conditions involving stress, pain-related manipulations, and infections [111,113,116-118,120,129-133]**.** Another complicating factor is that β-END levels show diurnal fluctuations in plasma as well as in the CSF [134,135]. The conclusion is that CSF β-END concentration mostly exceeds peripheral β-END, which excludes the possibility that central levels are a passive reflection of peripheral levels.

The functional correlation between CSF- and peripheral β-END is clear. With only one exception [136], all papers reporting a wide variety of experimental conditions, agree on the existence of complete dissociation between blood and CSF levels of β-END and on specific and different central *versus* peripheral regulatory mechanisms [116,120-122,128,129,137-140]. All studies since 1990 have consistently drawn the same conclusion: an intact BBB prevents the free exchange of β-END between plasma and CSF. Half-life values of β-END vary from 2 to 10 min in the peripheral circulation of rat and rabbit and between 20 and 50 min in the human circulation [115,141,142], while in the CNS, degradation of β-END hardly occurs at all [141,142]. The age–related changes throughout human life are also completely different: the peripheral levels show a parabolic peak at the age of about 50 years, while the CSF shows a steady decrease over the successive decades of life, down to less than 25% of peak at the age of 70+ [122].

Despite the general conclusion about separate central and peripheral control mechanisms, we have to keep in mind that the plasma and CSF compartments of β-END are not completely independent. Early experiments in the rabbit [115] already showed a steady but slow increase in CSF concentration after a single intravenous bolus injection of a radiolabeled marker. Starting after about 30 seconds, the radioactivity levels in the CSF increased up to 20-25% of the periphery after 60–90 minutes [115]. Apparently, peripheral β-END has some limited access to the CSF, but the delay is considerable. De Kloet *et al*[111] have discussed these possible pituitary-brain opiocortin transport mechanisms, consisting of vascular backflow via the terminal branches of the subependymal plexus [143], or backflow via the CSF and the pericapillary spaces of the median eminence [111] or uptake and retrograde axonal flow from the pericapillary spaces of the portal vessels [144]. In addition, membrane transporters of the organic anion-transporting polypeptide (Oatp) family may play a role in the transport of opioid peptides across the BBB and blood-CSF-barrier of the mammalian brain [145]. Such mechanisms may serve some long-term modulatory effect but are far too slow to affect changes in behavioral states, let alone the immediate physiological reactions to a painful stimulus.

### CSF β-END can be manipulated without affecting the peripheral levels

The strongest evidence that there is a separate central mechanism for β-END comes from the following experimental manipulations that induced elevated CSF levels without affecting peripheral concentration: electrical and chemical stimulation (10 Hz) of the ARH [146-148]; obesity [126] and ischemic attacks and strokes [125]; electroconvulsive shocks [149] and methadone maintenance schedules [150]; learning processes causing rapid CSF increases [151,152] and low levels of vasopressin affecting the release as well as the clearance of β-END from the CSF [116,153,154]. In these experiments in the rat brain, cisterna magna cannulation was shown to have differential effects on levels of β-END in plasma and CSF, which lasted for hours or even days [120].

In conclusion, for the purpose of the present review, we assume that the effects of peripheral levels of β-END on the CSF-levels are too limited and too slow to explain any of the direct brain and behavioral effects of central β-END. The extracellular pathways involved have been discussed by Banks and may possibly play a role in long-term treatment of Alzheimer’s disease or stroke [155,156].

Summarizing, we conclude that the available data suggest that central β-END effects on the CNS may occur in three successive stages using different time intervals. First, in a matter of milliseconds, β-END released from terminals and varicosities of the β-END-IR fibers may have immediate action on the neighboring receptive neuronal elements. Second, β-END, arriving with the flow of the CSF after seconds or only a few minutes, may influence a larger number of receptive brain areas for a longer period of time, partially sustaining the earlier neuronal release effects. Third, elevated peripheral levels of β-END slowly penetrate the CSF compartment and induce after a delay of 30 min or more, some increase in CSF concentration. However, these slow effects are perhaps modulatory and do not seem to play a role in the behavioral or pain-suppressing effects of β-END discussed below.

## β-END in the CSF

### Short- and long-distance volume transmission (VT)

**Figure 2:**
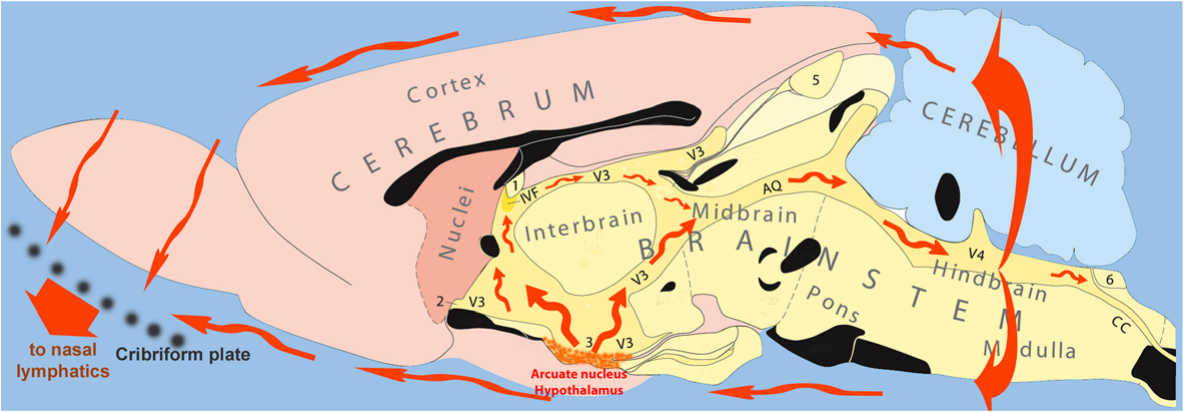
**Diagram showing the flow of CSF in the volume transmission of β-endorphin.** The main release site for β-END is the arcuate nucleus of the hypothalamus. The additional hindbrain site is located just ventral to no 6. The flow of the CSF (red arrows) traverses the aqueduct (AQ) to penetrate the mesencephalic periaqueductal gray before reaching the 4^th^ ventricle (V4), and along the ‘vagal-complex’ region. Both regions are important target areas for the flowing β-END. After leaving the ventricular system, the flowing CSF may affect superficial brain regions in the brainstem, hypothalamus and olfactory regions. A considerable part of the CSF and its contents eventually leaves the cranial cavity along the olfactory nerves penetrating the cribriform plate. The telencephalon is indicated in pink colours. The diencephalon (‘interbrain’) is coloured yellow, similar to the brainstem structures and the cerebellum is blue. Black structures show the location of fiber systems which cross the midline. Other symbols: numbers 1–6: circumventricular organs; cc: central canal of spinal cord; IVF: interventricular foramen, connecting the lateral and the 3^rd^ ventricles; V3: 3^rd^ ventricle. (The original figure was kindly provided by L.W. Swanson).

From the earliest reports, it has been recognized that POMC-fibers closely surround the ventricular spaces, traverse the subependymal layers and contain many varicosities. These may release their contents by non-synaptic release or exocytosis, either into the CSF or into the neighboring extracellular fluid (ECF) where it can influence receptive neurons locally [4,11,157,158]. This phenomenon has been denoted volume transmission (VT) [1-3,50,55,159,160]. It can work at short as well as long distances, guided by fiber tracts or by the flow of the CSF. Beta-END released from the ARH is in an excellent position to use long-distance VT for sending messages to a variety of brain areas using the caudally-directed flow of CSF (Figure 2). Beta-END was involved from the beginning in the development of the concept of volume transmission including the flow of CSF, as one of the distribution mechanisms [1-3,51,56,160-166]. Long-distance VT via the CSF has been implicated for a number of other neuromessengers: vasopressin and corticotropin-releasing hormone (CRH) [48,49], oxytocin [4], melatonin and gonadotropin-releasing hormone as well as for factors influencing food-intake [57-66].

*Strategic location of the ARH:* The median eminence of the hypothalamus is open to the portal vessels connecting the hypothalamus to the pituitary, without a BBB-ensheathment, but closed to the CSF. On the other hand, the ARH is open to the CSF in the third ventricle and in the subarachnoid space. Along the dorsolateral and ventromedial borders, the ARC is completely surrounded by relatively impenetrable barriers [79,167].These barriers consist mainly of tanycyte processes [167-172], which impede the diffusion of neuropeptides from the ARH into either the medially-located median eminence or the dorsolaterally-adjoining ventromedial hypothalamic nucleus [79]. This diffusion blockade in medial and lateral directions, combined with the open passage towards the third ventricle and the subarachnoid space [79,167,168,170,171], suggests that an activated group of POMC neurons in the ARH creates high levels of intranuclear POMC products that can only be released into the ventricular CSF (dorsomedial direction) or into the subarachnoid CSF (ventral direction). In addition to behavioral effects, opioid receptors are abundantly expressed in the proliferative zones of the fetal rat brain. They are likely targets for peptides distributed by CSF bulk flow and play a key role in modulating the mitotic activity and growth in neurogenic regions of the CNS [173]. The POMC neurons in the ARH are readily accessible for signals arriving within the CSF, because the flow along the ARH is slow due to the local absence of multiciliated cells [79]. On the other hand, β-END easily diffuses considerable distances in the brain [163]. The existence of a very long half-life of β-END in the CNS [142,174] supports the existence of these long-distance effects.

*Natural conditions rapidly increase central β-END levels:* There are behavioral conditions under which the β-END level increases rapidly in the CSF. Many animal and clinical studies have measured CSF and peripheral levels of β-END but observations were taken at 15 minutes after the experimental challenge [117,175]. In a limited number of studies, observations did start immediately, providing clear information about the speed of onset of the β-END reaction and/or effects [176,177]. Learning experiments using passive avoidance induced maximal CSF levels within 5 minutes [152,164]. After a variety of behavioral procedures*,* hypothalamic release of β-END started within a few minutes [151,178]. The amounts released, 20–40 ng per brain, are 8–10 times higher than the 2–5 ng needed to induce a behavioral effect after icv administration [151,178]. The effective dose after peripheral administration, on the other hand, is about 140 ng/rat [151], which is not inconsistent since only about 20% of the peripheral β-END may eventually reach the CSF by two hours after the injection [115,151].

*Artificially raised β-END in the CSF induces rapid effects:* Periventricular brain stimulation for pain relief in humans, resulted in an increase in β-END levels in the third ventricle CSF to a maximal value up to 20 times the basal level, within five minutes [176,177]. After icv administration of β-END, behavioral effects such as masticatory jaw movements, started within the first minute [179]. These rapid effects illustrate the capacity of the β-END system to release considerable amounts of the neuropeptide into the CSF, without delay. The following studies suggest that transport via the CSF is the best possible explanation for the observed effects. Chromaffin adrenal medullary cells produce and secrete several potential pain-reducing substances, including opioid peptides [180,181]. Yadid *et al.*[182] transplanted these cells into the subarachnoid space of the spinal cord of the rat and observed a marked reduction in pain behavior and showed the involvement of the central β-END mechanisms and the ARH in the observed analgesia, apparently via long-distance VT. In an another interesting study [183], genes were transferred into the meninges surrounding the spinal cord, causing pia mater cells to produce β-END. Clear analgesic effects were observed in an inflammatory model of persistent pain, apparently induced by β-END release into the CSF [183].

*Uptake of β-END from the CSF***:** For our hypothesis concerning long-distance VT, we also need to establish that specific ependymal and other cells, partially remote from the ventricular surface, are able to take up specific substances from the CSF. Such ependymal and neuronal elements are abundantly present throughout the ventricular system, including the lateral and fourth ventricles, and have been located in both forebrain (dentate area of the hippocampus, lateral septum, thalamus and hypothalamus) and a variety of brainstem areas, especially the raphe nuclei [51,53,161,184-186]. Retrograde flow mechanisms take care of the transport of substances like β-END, from the CSF towards the soma of neurons remote from the ventricles where they may elicit responses leading to changes in gene expression [51].

### Dendritic release of β-END?

The strategic situation of the POMC system shows striking similarities to that of the oxytocinergic (OT) system, which in mammals has two separate nuclei, the paraventricular hypothalamic nucleus bordering and freely accessing the third ventricle, and the supraoptic hypothalamic nucleus bordering and freely accessing the subarachnoid space, reviewed in [4]. The magnocellular oxytocinergic neurons release major amounts of OT via their dendrites [187-189], by inducing neighboring OT dendrites to join the OT release in the manner of a chain reaction and leading to a thousand fold increase in local concentrations of OT [190-192]. This questions how far dendritic release mechanisms of POMC neurons inside the ARH also play a role in the observed elevations of POMC levels in the nucleus as well as in the CSF. Direct evidence for such a release of β-END has not yet been obtained. This lack of data may just reflect the fact that the POMC population is more difficult to investigate than that of the OT-neurons, because of their location, density and their smaller size and other cellular characteristics. Although numerous contacts between, and the dense POMC innervation of POMC neurons, suggest that axonal/terminal release is the main mechanism for activating the POMC neurons as a group [92], some experimental findings make it implausible that interneuronal POMC-POMC interactions are purely axonal/terminal. First, it has been shown that dendrites in the ARH with and without spines may extend several hundreds of microns or even more than 1 mm in a dorsal or rostral direction from the nuclear borders [172,193]. Far away from the soma, thin fibers, probably axonal collaterals, arise from these POMC dendrites, suggesting that dendritic and axonal functions are not fully separate spatially or functionally [193]. POMC dendrites extending as far rostrally as the preoptic region were shown to release endocannabinoids to control local GABAergic inhibition [193], while in the opposite direction cannabinoids control the expression of β-END [194]. As dendrites from POMC-neurons extend and release substances far outside the anatomical border of the ARH itself, it seems worthwhile to study dendritic release mechanisms and their role in the interneuronal POMC interactions.

### Peptide release from POMC neurons: plasticity

In 1982, O’Donohue and Dorsa [113] mentioned that POMC neurons “secrete at least seven peptides which can be biotransformed to as many as five active peptides after release”. As mentioned before (section above), further processing of these neuropeptides may occur during axonal transport [70,82,83]. Fluctuations in functional plasticity have been discussed (see above) which may lead to considerable variations in connectivity, including serious disturbances in activity in specific neuronal networks. A similar influence has been described for β-END projections to the supraoptic nucleus [89]. In this case, however, manipulation of the pituitary-adrenal axis did not have much effect on mediobasal hypothalamic ACTH levels [195]. In addition, it has been observed that the amount of POMC, secreted into the CSF was 10–100 times larger than the amount of ACTH or β-END [196,197]. Energy homeostasis, with leptin playing a crucial role [198-200], caused great variability in the balance between POMC and its derivatives, ACTH or α-MSH, in the ARH, which is richly provided with leptin receptors. Variations in the CSF-levels turned out to be more pronounced than in the ARH brain tissue itself [198]. Apparently, the balance between diverse POMC products is regulated and hormonal signals controlling food intake are involved in this effect. Finally, a mutually balancing mechanism has been proposed between β-END on the one hand and the melanopeptides (ACTH and α-MSH) on the other, in that continuous icv infusion stimulates the development of tolerance as well as increased production of the counterbalancing peptides in the ARH [201,202].

From these data, we conclude that plasticity in the peptide cocktail of the POMC neurons does indeed occur. In addition to tolerance effects, hormonal factors, so far mainly controlling energy homeostasis, may induce changes in the composition of the peptide cocktail which may have serious consequences for the different activation/inhibition patterns in the projection areas of the opiocortin neurons. Interestingly, such plastic changes are even region-specific to some extent, because certain hormonal conditions induced changes in local hypothalamic β-END levels only, without affecting other hypothalamic nuclei [203]. In addition, and very importantly for the purpose of the present review, the CSF levels of POMC and its derivatives are not a mere reflection of the intracellular neuropeptide balance, but are controlled by specific mechanisms. See Pritchard and White [200] for an extensive review of the cellular mechanisms involved.

### Functional subgroups of β-END neurons

Interestingly, many POMC neurons participate in a dense local network resulting in POMC-POMC synapses inside parts of the ARH [67,68,70,72,73,80,90,92,95]. As suggested by a similar mechanism for oxytocin reviewed by Veening *et al*[4] , such contacts may synchronize the activities of the POMC neurons forming one or more functional units, allowing them “to perform coherently as a robust processing unit” [204], for instance to provide the considerable amounts of POMC needed to elevate the CSF levels.

Anatomically, the population of POMC neurons does not form a single functional unit. As mentioned, there are two groups of neurons, one located in the ARH and other in the NTS. These sources have widely diverging projections: the ARH mainly to mesencephalic, hypothalamic, limbic and forebrain areas, and the NTS mainly to the caudal brainstem and spinal cord [106,107]. Overlapping projections from both groups were only observed in a number of brainstem areas, like locus coeruleus and parabrachial nucleus, with a distinctive pattern for both projections in the latter [106].

But even the POMC neurons in and around the ARH do not seem to be a single homogeneous group either anatomically or functionally. Retrograde tracer studies combined with POMC-immunocytochemical staining are relatively scarce but where available they show that only a limited portion of the POMC neurons become labeled after each tracer injection, with several indications for a topographical organization of the origins. Such retrograde double-labeling studies showed that only about 20% of the POMC neurons project to the preoptic area [101,103]. Labeled neurons were bilaterally distributed throughout the rostrocaudal extent of the ARH, with a peak at the middle levels [101], roughly coinciding with subgroup 1 described in [68]. After more dorsal preoptic injections, the pattern of labeled POMC-neurons tended to shift to the lateral parts of the ARH [101]. Chronwall [205] observed a similar distribution after preoptic injections, without rostrocaudal differentiation, but ARH neurons projecting into the PAG were observed more dorsolaterally, with only a small percentage of neurons projecting to both brain areas. Yoshida and Taniguchi [96] demonstrated that most of the PAG-projecting neurons were concentrated in the rostral three-fifths of the ARH, about 20% of them containing β-END. Sawchenko *et al.*[94] showed that about 600 basomedial hypothalamic neurons could be stained for ACTH, and about 40% of them, mostly in the ventral part of the ARH, projected to the paraventricular hypothalamic nucleus. More recently, Douglas *et al.*[89] found that in the rat, the number of POMC-producing neurons in the ARH increases considerably during pregnancy, especially caudally, whereas the retrogradely-labeled ARH neurons projecting to the supraoptic nucleus, contained about 20% β-END. All of these tracer experiments suggest that each of the projections arises from a limited number of β-END neurons, and additional quantitative experiments are required to elucidate this aspect.

Estrogen receptivity is another factor differentiating between 4-20% of the β-END neurons from the majority of the neuronal population [206-208]. These neurons have been observed equally dispersed over the total β-END population and their number may vary upward or downward by about 50% after manipulation of estrogen levels [207,209]. It was known already that the distribution pattern of β-END-fibers in the medial part of the medial preoptic nucleus show sexually dimorphic differences [210] while castration of photostimulated male hamsters induces >50% increase in β-END levels in the mediobasal hypothalamus [203].

In summary, it is clear that β-END neurons ‘never walk alone’, anatomically and functionally, but operate in subgroups distributed over different regions of the ARH. Projections to specific brain regions may arise from different subgroups of β-END neurons, since there are multiple indications for functional differentiation, e.g. estrogen receptivity**,** as well as topographical organization in the distribution of the fibers originating from different parts of the ARH. Additional experiments, combining various retrograde tracers with immunological or *in situ* hybridization techniques, are required to elucidate the functional topographical organization of the mediobasal hypothalamic β-END neurons in more detail.

### Summary and questions

From the preceding sections we conclude that β-END can be released into the CSF in sufficient quantities to induce physiological and/or behavioral effects. The β-END neurons are located in the mediobasal hypothalamus with, on the one hand, interconnections which permit them to function as sub groups and on the other an internal topographical organization, enabling them to send messages to specific targets. Dendritic release from POMC neurons in the ARH probably occurs but its contribution relative to axonal release from varicosities and terminals, remains to be investigated. Production and release of POMC and its derivatives is under control of external factors such as leptin, for energy homeostasis. This implies that differential messages can be released into the CSF and/or neuronal target areas participating in the involved neuronal circuitry. From this summary, it is clear that quite a few questions remain to be answered. What are the input–output relationships among and within the subgroups of POMC neurons? Do all POMC subgroups contribute to the release into the CSF? Which external factors influence the activity and productivity of POMC neurons? Do all POMC neurons show the same sensitivity for factors like leptin or GABA-ergic inhibition via the preoptic region?

While several additional questions can be raised, those mentioned can all be studied by combinations of readily available techniques.

## Long-Distance VT effects of β-END

### Downstream location of β-END receptive brain areas

β-END reacts with at least two types of opioid receptors: mu and delta [211]. The location of these receptors has been determined using a variety of methods, ranging from binding studies to recent mRNA techniques [212-228]. The distribution patterns are quite different for the two receptor types and a full discussion would go far beyond the scope of the present review. Furthermore, the distribution patterns vary widely between different species [211,221].

There is a so-called ligand-receptor mismatch, where the distribution of immuno-labeled terminals is different to the distribution of the relevant receptors [229,230]. In fact, the occurrence of this β-END terminal-receptor mismatch was the starting point for the concept of VT as developed by Agnati and Fuxe and coworkers [1-3,50,52-56,159,161,165,166,230]. Some of the important areas where β-END has an effect will be mentioned. MacMillan *et al*[148], studied the destination of β-END released by low-frequency electrical stimulation of the ARH. They concluded that “the cerebrospinal fluid is an important mechanism of the transport of β-END” and that in this way “β-END will affect brain function in a widespread or global manner”. The many circumventricular β-END-receptive areas show considerable differentiation [148]. The periaqueductal gray (PAG) and the vagus complex are among the first brain areas exposed to elevated levels of β-END released into the CSF. These brain areas are highly receptive and play a prominent role in pain-regulation, sexual behavior and food intake, as discussed below. The distance between these brain areas and release from the ARH is so small that volume transmission may take effect almost immediately.

In the human brain, specific endorphinoreceptive neurons have been described in the deeper layers of the cingulate and frontal cortices, which are densely ensheathed by β-END terminals [231]. These layers are easily accessible for β-END in CSF. The analogous areas in the rat brain were shown previously to be highly opioid receptive [212]. In between the many densely-innervated brain areas, it is remarkable that large parts of the brain, like most of the neocortex, striatum and hippocampus, but also a few specific hypothalamic nuclei like the ventromedial hypothalamic nucleus and the mammillary nuclei, are virtually devoid of any opiocortin innervation [211].

Receptor density alone may not be the best indicator for the size of the expected effect, behaviorally or metabolically, because of possible indirect effects. Ableitner and Schulz [179] measured the local cerebral glucose utilization, as a correlate of neuronal activity, after icv administration of β-END in the rat. The most marked increases were observed in the hippocampal formation despite the lack of opiocortin innervation, especially the ventral components, and in some closely-related limbic areas. Thalamic nuclei and the caudate-putamen complex, harboring high densities of mu and delta receptors, respectively, hardly reacted to the icv-administration.

It has been shown by Herbert and his coworkers [232-235] that the behavioral effects of local β-END infusion may be very subtle, affecting only some specific behavioral transitions. Such local effects can elucidate the specific effects of β-END but they easily get hidden when focusing only at the general behavioral effects of the neuropeptide. β-END is known to induce euphoria and to have rewarding and reinforcing properties [236]. Numerous recent reviews have discussed the involvement of mu receptors in the ‘liking’ and ‘wanting’ aspects of food reward [237-247]. The functional relationship between the rewarding aspects of sexual behavior and the involvement of opioids are also supported in a series of papers and reviews [15,248-259]. The bidirectional interactions between the opioid systems, including β-END, and the mesolimbic (and incerto-hypothalamic -dopaminergic systems form neural substrates for the reward effects of eating and sexual behavior and can be considered as crucial components of the mechanisms involved in motivational drives and goal-directed behavior. The motivational effects of numerous neuroactive substances reflect their inhibitory or excitatory action on this dopaminergic reward system, extending between the ventral tegmental area and the nucleus accumbens. It has been stated that “The (induction of a) reward state in males and females is mediated by opioids and the medial preoptic area of the anterior hypothalamus is a crucial site for sexual reward” [252]. In addition, β-END also plays a role in addiction because of its mutual modulatory relationships with the mesolimbic dopaminergic system [260-265].

### Is ‘addressing’ of β-END used for regional specific effects?

The findings discussed in the preceding sections raise interesting possibilities. If POMC neurons function in separate groups, then messages may be released into specific areas of the ventricular system and affect particular CNS sites responsible for physiological/behavioral reactions. There is evidence to support this. Experiments show that when a specific messenger is released into the ventricular system, the location plays an important role in the resulting effects. For example, administration of 5-HT into the lateral but not the 3^rd^ ventricle stimulated female sexual behavior in rats [266] and oxytocin administered into the lateral ventricle but not into the 3rd ventricle, suppressed lordosis [267]. On the other hand, β-END administered into the lateral ventricle facilitated lordosis but inhibition occurred after 3^rd^ ventricular administration [268]. Similar contrasting effects were observed after lateral ventricle *versus* cisternal administration of lysine-vasopressin [269,270]. The inhibitory effects of relaxin on the milk ejection reflex as well as the haemotensive responses elicited by icv administration, were completely different depending on the site of injection [271,272]. These findings show that the site of application into the CSF is very important. To study the control of food intake, many additional experiments were performed, aiming at the relative contribution of fore- and hindbrain areas, with specific administration of a wide variety of substances into either the 3^rd^ or the 4^th^ ventricle, combined with or without occlusion of the cerebral aqueduct. It is beyond the scope of the present review to discuss these in detail, but it is clear that factors flowing in the CSF will reach the 4^th^ ventricle region and influence brainstem areas like area postrema and the solitary complex that control food intake. Hypothalamic and hindbrain melanocortin receptors play an important role [273,274] and the hindbrain relationships with the POMC neurons have been extensively explored [275-279].

In addition to the contributions of the hypothalamo-medullary projections, however, the flowing CSF deserves more attention as a medium for sending messages into the brainstem. ARH-activation induces more than 15-fold increases in β-END in the CSF [176], and the CSF transports these products to the effective sites in the mesencephalic central gray region [146,147,177,280-282]. On the other hand, deep brain stimulation in the periventricular gray region, in patients suffering chronic pain, resulted in two- to threefold increases in CSF levels of β-END in the rostral horn of the lateral ventricle [177]. This raises the question how far this increase is the result of terminal release of β-END from the mesencephalic opiocortin fibers. It is possible that the electrodes also activated the β-END cell bodies antidromically, inducing additional dendritic release in the arcuate nucleus.

Finally, since POMC seems to be released into the 3^rd^ ventricular CSF in amounts at least 10 times larger than the peptide derivatives [196,197], it might be rewarding to study volume transmission via the CSF towards the dorsal and ventral hindbrain, as an active mechanism to support neuronal transmission of information. Without exception, all authors of above cited papers conclude that different populations of neurons, bearing different types of receptors, are responsible for the differential effects after local ventricular administration. Concerning β-END, having different effects on sexual behavior, food intake and pain regulating mechanisms, it is clear that widely divergent brain areas play a major role, from amygdala and rostral hypothalamic sites to midbrain and caudal brainstem sites. The quality of messages, delivered via the CSF, would improve considerably if substances can be delivered at optimal locations in the ventricular system. The high density of POMC-fibers, surrounding the ventricular system, suggests that they are in an excellent position to do so.

There is no direct experimental evidence available that β-END can be released by specific opiocortin terminals at other ventricular sites than the lower part of the 3^rd^ ventricle, surrounded by the arcuate nucleus. Maybe the use of larger mammals, like sheep or even cows, is necessary to obtain experimental evidence for an addressing system with specific behavioral effects. Using sheep, has turned out to be very fruitful in studies concerning release and circulation patterns of melatonin [62,64,66] and GnRH [57,63,283,284]. The most convincing evidence would be obtained if it could be shown that the distribution of POMC-concentrations over the different parts of the ventricular system are not identical under different conditions, like feeding, sexual behavior, or suffering from a painful stimulus.

### The periaqueductal gray and other regions as targets for the β-END-flow

The population of POMC neurons is apparently able to produce massive amounts of β-END which is released into the 3^rd^ ventricle, to reach the cerebral aqueduct via the CSF flow and to influence the periaqueductal gray (PAG) region of the mesencephalon [146,148,176]. This region is closely located to the CSF source, the ARH, and is traversed by numerous POMC fibers [11,68,70,73-76,89,90,93,210]. We have discussed previously that neuronal connections with axonal varicosities may mutually support each other via short-term synaptic transmission and via mid-term (up to a few minutes) transport of CSF messages for brain areas, especially those surrounding the ventricular system, as fast axonal release of β-END from the varicosities in the PAG will penetrate the intercellular space between the PAG-neurons. Slightly later β-END released in the ventral part of the 3^rd^ ventricle will arrive with the flow of the CSF to the PAG. When local levels start to diminish after the instantaneous release, the elevated CSF levels arriving from the ARH start diffusing into the surrounding tissue to sustain and prolong the local behavioral effects of elevated β-END levels. This enables the target circuitry to be programmed in a flexible way: a different cocktail of neuropeptides could induce different functionalities of the circuit, by modifying simultaneously a relevant set of parameters.

## Conclusions

In summary the evidence reviewed here suggests that the combination of axonal release of β-END in specific brain areas and its transmission via the CSF vary according to brain region. The varicose fibers surrounding the ventricular system allow short- as well as long-distance VT. The axonal messages of subgroups of β-END neurons are targeting specific destinations. The CSF-messages, released from the mediobasal hypothalamus, will arrive with some delay at the downstream brain areas, supporting and extending the effects of axonal messages.

We conclude that β-END, released into the flowing CSF, may have effects on distant brain regions where they affect a variety of behaviors relating to reward mechanisms and motivational and mental states, and lead to stress-reduction and homeostatic balance.

## Abbreviations

5-HT, Serotonin; ACTH, Adreno-corticotropic hormone; α-MSH, α-melanocyte-stimulating hormone; ARH, Arcuate nucleus of the hypothalamus; β-END, Beta endorphin; BBB, Blood–brain-barrier; CNS, Central nervous system; CRH, Corticotropin releasing hormone; CSF, Cerebrospinal fluid; GABA, γ-aminobutyric acid; GnRH, Gonadotropin releasing hormone; icv, Intracerebroventricular (administration); NTS, Nucleus of the solitary tract; OT, Oxytocin; PAG, Periaqueductal gray region of the midbrain; POMC, Pro-opio-melano-cortin containing neurons or fibers; VT, Volume transmission.

## Competing interests

The authors declare that they have no competing interests.

## Authors’ contributions

JGV: 80%, composition, contents, writing; HB: 15%, composition, discussion, improvements and corrections; POG: 5%: discussion and general improvements; All authors have read and approved the final version of the manuscript.
